# Prolonged evolution of the human B cell response to SARS-CoV-2 infection

**DOI:** 10.1126/sciimmunol.abg6916

**Published:** 2021-02-23

**Authors:** Mrunal Sakharkar, C. Garrett Rappazzo, Wendy F. Wieland-Alter, Ching-Lin Hsieh, Daniel Wrapp, Emma S. Esterman, Chengzi I. Kaku, Anna Z. Wec, James C. Geoghegan, Jason S. McLellan, Ruth I. Connor, Peter F. Wright, Laura M. Walker

**Affiliations:** 1 Adimab LLC, Lebanon, NH 03766, USA.; 2 Division of Infectious Disease and International Health, Dartmouth Hitchcock Medical Center, Lebanon, NH 03756, USA.; 3 Department of Molecular Biosciences, The University of Texas at Austin, Austin, TX 78712, USA.; 4 Adagio Therapeutics, Inc., Waltham, MA 02451, USA.

## Abstract

A comprehensive understanding of the kinetics and evolution of the human B cell response to SARS-CoV-2 infection will facilitate the development of next-generation vaccines and therapies. Here, we longitudinally profiled this response in mild and severe COVID-19 patients over a period of five months. Serum neutralizing antibody (nAb) responses waned rapidly but spike (S)-specific IgG^+^ memory B cells (MBCs) remained stable or increased over time. Analysis of 1,213 monoclonal antibodies (mAbs) isolated from S-specific MBCs revealed a primarily de novo response that displayed increased somatic hypermutation, binding affinity, and neutralization potency over time, providing evidence for prolonged antibody affinity maturation. B cell immunodominance hierarchies were similar across donor repertoires and remained relatively stable as the immune response progressed. Cross-reactive B cell populations, likely re-called from prior endemic beta-coronavirus exposures, comprised a small but stable fraction of the repertoires and did not contribute to the neutralizing response. The neutralizing antibody response was dominated by public clonotypes that displayed significantly reduced activity against SARS-CoV-2 variants emerging in Brazil and South Africa that harbor mutations at positions 501, 484 and 417 in the S protein. Overall, the results provide insight into the dynamics, durability, and functional properties of the human B cell response to SARS-CoV-2 infection and have implications for the design of immunogens that preferentially stimulate protective B cell responses.

## Teaser

Longitudinal analysis of the human B cell response to SARS-CoV-2 infection reveals prolonged antibody evolution.

## INTRODUCTION

Severe acute respiratory syndrome coronavirus 2 (SARS-CoV-2), the causative agent of the coronavirus disease 2019 (COVID-19) pandemic, has infected over 75 million people and claimed over 1.5 million lives in just 12 months. Although vaccines have been developed and deployed at an unprecedented pace, the protection afforded by these vaccines may be short-lived due to waning serum antibody titers and/or the emergence of SARS-CoV-2 strains that evade vaccine-induced immunity (*1*–*7*). A detailed characterization of B cell responses induced by natural infection will provide key insights into the durability and breadth of protective immune responses and may facilitate the design of next-generation COVID-19 vaccines and therapies.

Numerous studies have shown that serum neutralizing antibody (nAb) responses targeting the SARS-CoV-2 spike (S) protein appear within two weeks following symptom onset and persist for several months (*8*–*11*). Although high titers of serum nAbs are required for sterilizing immunity, memory B cells (MBCs) rapidly re-activate following a secondary infection and can contribute to protection against severe disease or death (*12*). Human MBCs can differentiate into antibody-secreting cells to provide an immediate source of serum antibody or they can enter secondary germinal centers (GCs) to re-diversify their B cell receptors (BCRs) in response to evolving or antigenically related pathogens (*12*). Despite the potential importance of MBCs in protection against secondary COVID-19, the characteristics of the MBC response elicited by primary SARS-CoV-2 infection remain incompletely defined. Here we present an analysis of the kinetics and evolution of this response, as well as an in-depth characterization of the mAbs encoded by these B cells.

## RESULTS

### Longitudinal analysis of serum binding and neutralizing titers

**Fig. 1:**
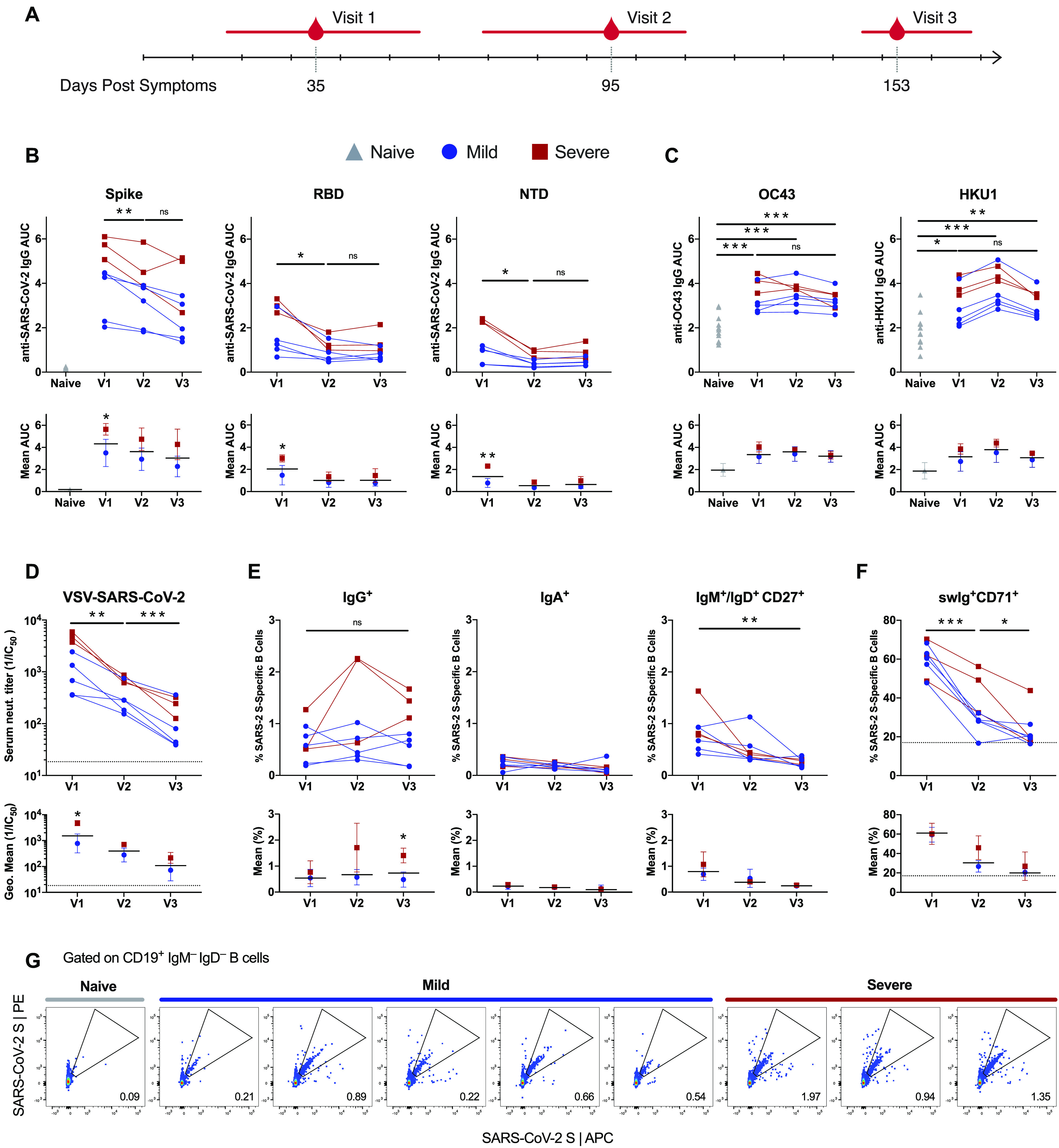
Longitudinal analysis of anti-SARS-CoV-2 serum and memory B cell responses. (A) Blood samples were collected at the indicated time points post-symptom onset. The values underneath the timeline indicate medians and the red bars indicate ranges. (B-C) Serum IgG binding to SARS-CoV-2 (B) and endemic β-CoV (C) S protein antigens, as assessed by ELISA (top). Mean IgG binding titers in donors with mild and severe COVID-19 (bottom). Twelve pre-pandemic naive donor samples are included as controls. Error bars denote standard deviation and black bars indicate means. (D) Serum VSV-SARS-CoV-2 neutralizing titers for each donor at the three sampling timepoints (top). Geometric mean serum neutralizing titers in mild and severe donor samples (bottom). Error bars denote geometric standard deviation and black bars indicate geometric means. The dotted line indicates the lower limit of detection. (E) Frequencies of SARS-CoV-2 S-specific IgG^+^, IgA^+^, and IgM^+^ and/or IgD^+^CD27^+^ B cells at each sampling time point (top). Mean frequencies of SARS-CoV-2 S-specific B cells expressing the indicated isotype in mild and severe donors (bottom). Error bars denote standard deviation and black bars indicate means. (F) Proportion of SARS-CoV-2 S-specific swIg^+^ B cells that express CD71 at each sampling time point (top). Mean frequencies of SARS-CoV-2 S-specific swIg^+^CD71^+^ B cells in mild and severe donors (bottom). Error bars denote standard deviation and black bars indicate means. The dotted line indicates the level of CD71 expression on swIg^+^ B cells in pre-pandemic donor samples. (G) Representative gating for SARS-CoV-2 S-specific B cells. The gated populations were single cell sorted for mAb cloning. Statistical comparisons between naive and convalescent donors were made by two-sided one-way Welsh ANOVA for unpaired samples with Dunnett’s T3 multiple comparisons test. Statistical comparisons between timepoints among convalescent donors were performed by two-sided two-way ANOVA for paired samples with Tukey’s multiple comparisons test. Statistical comparisons between mild and severe convalescent donor cohorts at each timepoint were performed by two-sided two-way ANOVA with Sidak’s multiple comparisons test. *, P < 0.05; **, P < 0.01, ***, P < 0.001; ns, not significant; AUC, area under the curve.

We collected longitudinal serum and peripheral blood mononuclear cell (PBMC) samples from a cohort of eight COVID-19 convalescent donors who experienced severe (n=3) or mild (n=5) disease. The first blood sample (Visit 1) was drawn a median of 35.5 days following the onset of symptoms to allow for an assessment of the early B cell response to SARS-CoV-2 infection. The second (Visit 2) and third (Visit 3) blood samples were collected a median of 95.5- and 153.5-days post-symptom onset, respectively, to evaluate the long-lived memory B cell response (Fig. 1A and Table S1). We first assessed serum immunoglobulin G (IgG) binding to a panel of SARS-CoV-2 and endemic beta-coronavirus (β-CoV) S protein antigens by ELISA (Fig. 1B, C). Consistent with prior reports, we observed variable levels of anti-SARS-CoV-2 serum binding antibodies across donors and overall higher titers in the donors with severe disease (*8*, *13*). Between Visits 1 and 3, the levels of anti-S, anti-receptor-binding domain (RBD), and anti-N-terminal domain (NTD) antibodies declined in most donors (Fig. 1B). Interestingly, all of the COVID-19 convalescent donors displayed significantly elevated serum IgG binding to the S proteins of seasonal β-CoVs, OC43 and HKU1, relative to pre-pandemic samples at all three sampling time points, suggesting recruitment of broadly cross-reactive antibodies into the response (Fig. 1C).

We next measured nAb activity in the serum samples using a previously described pseudovirus reporter assay based on vesicular stomatitis virus bearing SARS-CoV-2 S (VSV-SARS-CoV-2) (*14*). Serum neutralization of VSV-SARS-CoV-2 and authentic SARS-CoV-2 are strongly correlated, demonstrating the suitability of this system for high-throughput analysis of viral infection inhibition (*15*, *16*). All donors mounted a serum nAb response by Visit 1, and the donors with severe disease displayed overall higher neutralizing titers relative to donors with mild disease (Fig. 1D). However, we observed a significant decline in serum neutralizing activity over time in all donors. The geometric mean half-maximal neutralizing titer in this group of eight donors decreased from 1,526 to 125 between Visit 1 and Visit 3, respectively (Fig. 1D). In summary, we observed a progressive decline in serum binding and neutralization titers over ~120 days in our donor cohort, but neutralizing activity was still detectable in all donor samples at the last sampling time point.

### Kinetics of the SARS-CoV-2 S-specific memory B cell response

We next evaluated the kinetics and phenotypic diversity of the MBC response to SARS-CoV-2 S over the ~120-day sampling period. To identify SARS-CoV-2 S-reactive MBCs, we stained PBMC samples from the eight donors with a panel of B cell surface markers (CD19, CD27, IgG, IgA, IgM, IgD and CD71) and a fluorescently labeled, prefusion stabilized SARS-CoV-2 S protein (*17*) (Fig. S1A). Exploratory analysis showed that antibody-secreting cells (ASCs), defined as CD19^+^CD27^+^CD38^+^ B cells, had contracted to background levels by the first sampling time point in all donors (Fig. S1B, C). We detected SARS-CoV-2 S-specific IgG^+^ MBCs in all donors at Visit 1, and the frequencies of MBCs either increased or stabilized by Visit 3, providing evidence for continuous MBC formation over a period of several months (Fig. 1E). Additionally, in all donors, over 50% of SARS-CoV-2 S-specific B cells expressed the B cell activation/proliferation marker CD71 at Visit 1 and this marker remained elevated over background levels at Visit 2, further supporting robust activation of MBCs (Fig. 1F). Notably, this result is consistent with previous studies of B cell responses to Ebola, yellow fever virus, and SARS-CoV-2, which also documented prolonged expression of CD71 on the surface of antigen-specific B cells following infection or vaccination (*18*–*20*). Consistent with the higher serum antibody titers observed in donors with severe COVID-19, these donors also showed a higher frequency of S-specific IgG^+^ MBCs at the last sampling time point relative to those with mild disease. In contrast, SARS-CoV-2 S-specific IgM^+^ and IgA^+^ MBCs were present at variable levels at the earliest sampling time point and declined to low or undetectable levels by Visit 3 in most donors (Fig. 1E). Thus, although serum neutralizing titers declined substantially over the study period, IgG^+^ MBCs continued to accumulate for several months following the resolution of clinical disease.

### Evolution of the SARS-CoV-2 S-specific memory B cell response

**Fig. 2:**
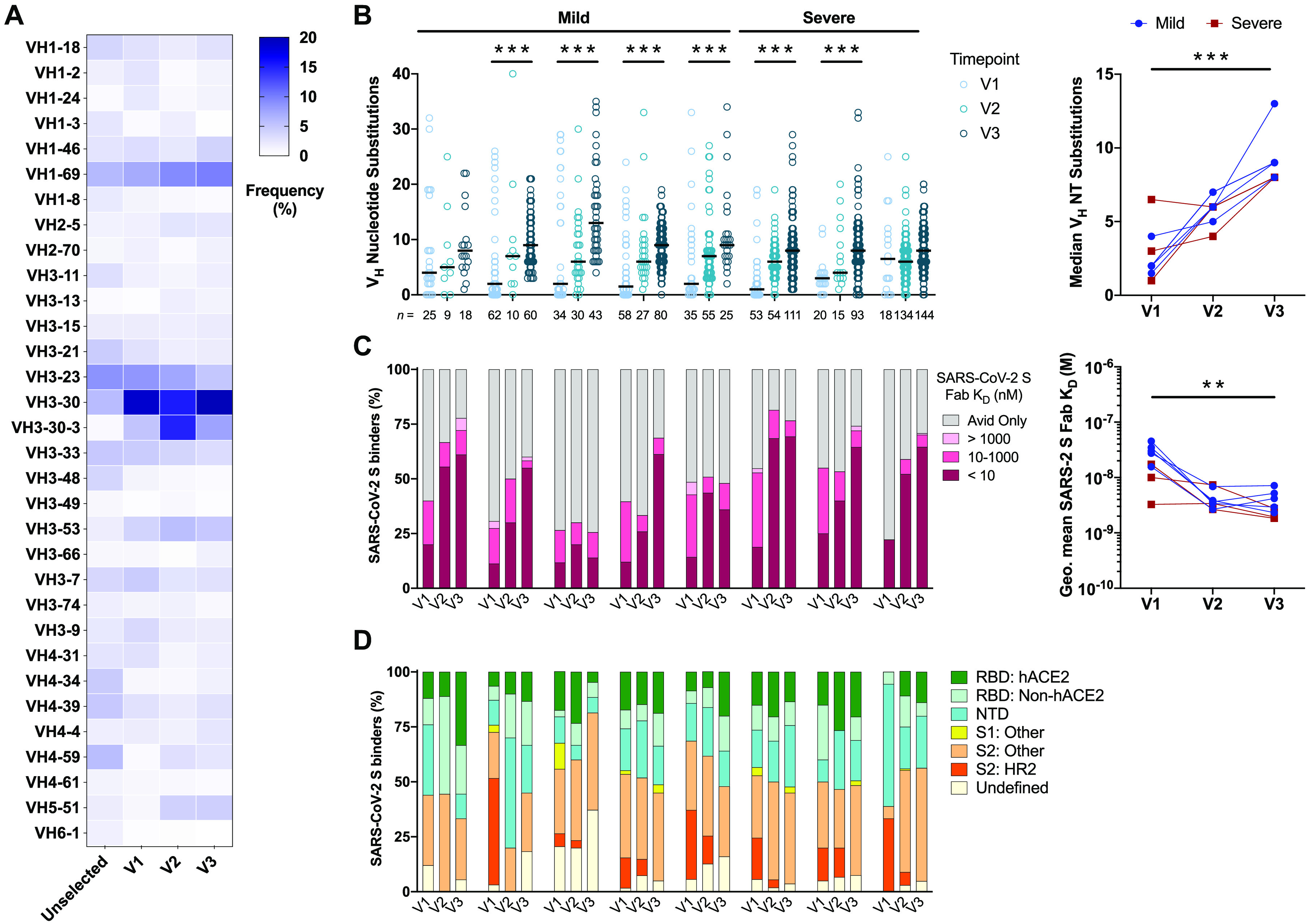
SARS-CoV-2 S-specific antibody sequencing and binding characteristics. (A) VH germline gene usage of SARS-CoV-2 S-specific mAbs isolated at each sampling time point. VH germline gene frequencies of unselected human MBCs (Unselected) were obtained from high-throughput sequencing studies and shown for comparison (*44*). (B) SHM loads of SARS-CoV-2 S-specific mAbs isolated from each donor at Visits 1-3 (left), with the number of mAbs analyzed per timepoint displayed below the axis. Statistical comparisons were performed by two-sided Kruskal-Wallis tests with Dunn’s multiple comparisons test. Median number of VH nucleotide substitutions in SARS-CoV-2 S-specific mAbs isolated from each donor at Visits 1-3 (right). Statistical comparisons were made by two-sided Friedman test with Dunn’s multiple comparisons test. Black bars indicate medians. (C) Proportion of SARS-CoV-2 S-specific mAbs isolated at each time point with the indicated Fab binding affinities. The avid-only group contains mAbs that bound to SARS-CoV-2 S in an avid but not monovalent orientation (left). Geometric mean Fab binding affinities of SARS-CoV-2 S-specific MAbs isolated from each donor at Visits 1-3 (right). MAbs that did not display detectable Fab binding are excluded from this analysis. Statistical comparisons were made by two-sided Friedman test with Dunn’s multiple comparisons test. (D) Proportion of SARS-CoV-2 S-specific mAbs isolated at Visits 1-3 that target the indicated antigenic sites. RBD: hACE2, RBD-directed and hACE2 competitive; RBD: non-hACE2, RBD-directed and hACE2 non-competitive; S1: Other, S1-reactive but non-reactive with isolated NTD or RBD proteins; S2: Other, reactive with S2 and SΔHR2; S2: HR2, reactive with S2 but not SΔHR2. NT, nucleotide. **, P < 0.01, ***, P < 0.001.

To further dissect the MBC response to SARS-CoV-2 S, we single-cell sorted between 23 to 368 SARS-CoV-2 S-reactive switched immunoglobulin (swIg)^+^ MBCs per donor at each sampling time point and cloned a total of 1,213 paired heavy- and light-chain genes as full-length IgG1s (Fig. 1G and Fig. S2). Index sorting analysis showed that the majority of SARS-CoV-2 S-specific IgG^+^ and IgA^+^ MBCs expressed the canonical MBC marker CD27 at all sampling time points (Fig. S3). Sequencing studies revealed high clonal diversity in the donor repertoires at all three time points studied, with 0-30% of clones belonging to expanded clonal lineages (Fig. S4A). In agreement with prior studies of early antibody responses to SARS-CoV-2 infection (*13*, *21*), we observed a significant over-representation of mAbs utilizing the VH3-30, VH3-30-3, and VH3-53 germline gene segments in the S-specific repertoires relative to naïve donor repertoires at each sampling time point (Fig. 2A). Across all eight donors, the median level of somatic hypermutation (SHM) was low at the first sampling time point—with 15-42% of mAbs from each donor lacking somatic mutations—and increased gradually over the ~120-day study period (Fig. 2B). By Visit 3, the median number of nucleotide substitutions in the variable region of the heavy chain (VH) was 9 and 8 in donors with mild and severe disease, respectively (Fig. 2B). Furthermore, the mAbs isolated at all three timepoints showed an enrichment for replacement mutations over silent mutations in complementarity-determining regions (CDRs) 1 and 2 compared with framework regions (FRs) 1–3 at each timepoint, providing evidence of antigen-driven selection (Fig. S4B). Consistent with their increased SHM loads, the mAbs isolated from most Visit 2 and 3 samples displayed higher Fab binding affinities to SARS-CoV-2 S relative to the mAbs isolated from Visit 1, indicative of on-going affinity maturation (Fig. 2C and Fig. S4C).

To investigate whether SARS-CoV-2 S-specific antibody immunodominance hierarchy shifts over time, we evaluated the antigenic sites targeted by the 1,213 mAbs isolated from all three time points using a panel of SARS-CoV-2 subunits and domains [RBD, NTD, Subunit 1 (S1), and a recombinant Subunit 2 (S2) that contains pre-fusion stabilizing mutations (*22*)]. We also tested the RBD-directed mAbs for competition with the human entry receptor ACE2 (hACE2) in a sandwich-binning biolayer interferometry (BLI) assay. Although all the donor repertoires displayed broad antigenic site coverage, S2-directed mAbs dominated many of the donor responses at all three sampling time points (Fig. 2D). Further mapping of these mAbs using an S variant lacking the second heptad repeat (SΔHR2) revealed that the S2 binders recognized epitopes both within and outside of the HR2 region (Fig. 2D). Finally, in most donors, we observed similar antigenic site distribution over time, suggesting that antibody immunodominance hierarchy was relatively stable during maturation of the MBC response to SARS-CoV-2 infection (Fig. 2D).

**Fig. 3:**
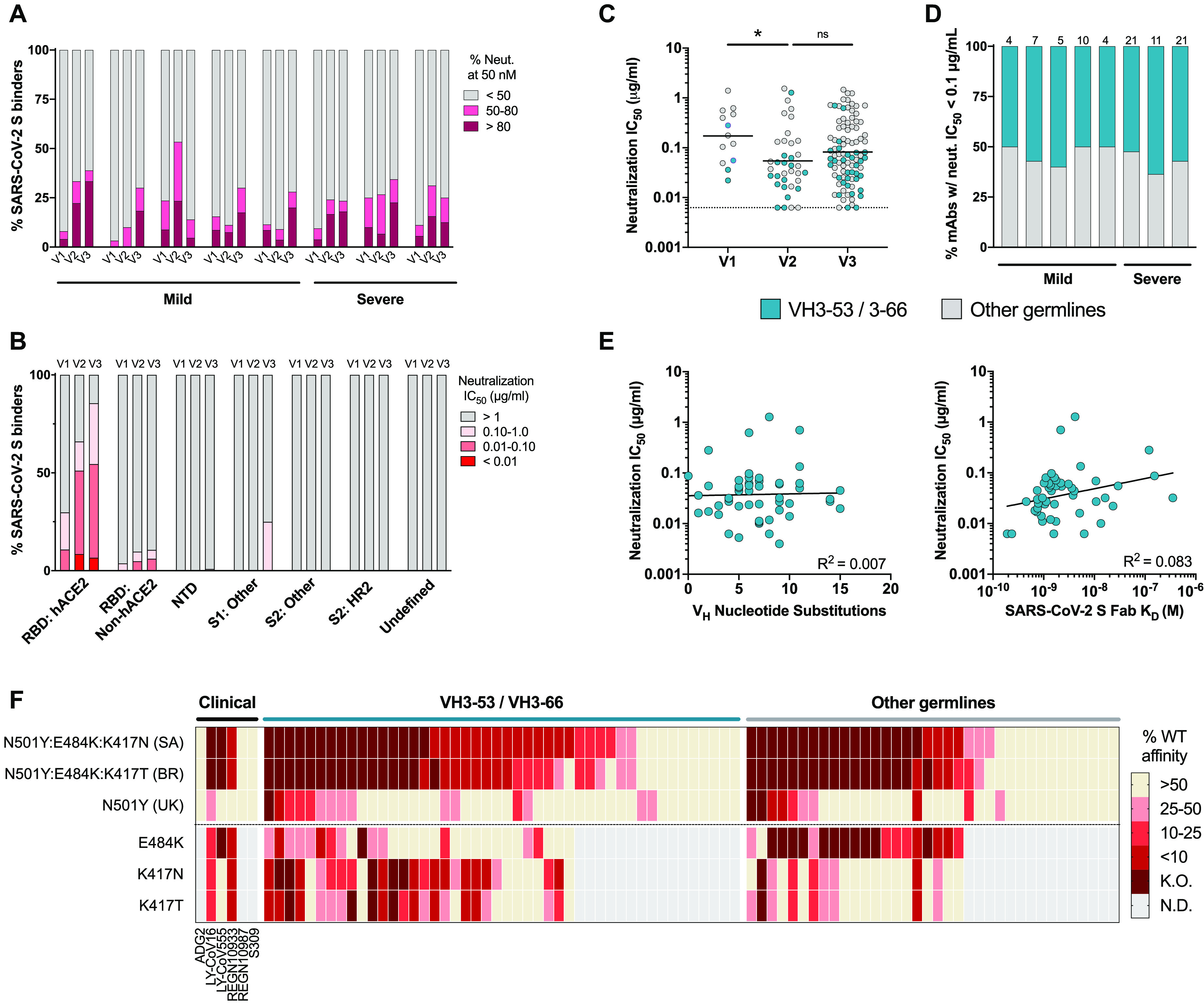
Longitudinal analysis of the neutralizing antibody response to SARS-CoV-2. (A) Proportion of SARS-CoV-2 S binding mAbs isolated at Visits 1-3 with the indicated level of VSV-SARS-CoV-2 neutralizing activity at a concentration of 50 nM. (B) Proportion of mAbs targeting each of the indicated antigenic sites that display the indicated neutralization potencies. (C) VSV-SARS-CoV-2 neutralization IC_50_s of mAbs isolated at Visits 1-3 that displayed >80% neutralizing activity in the initial screen. MAbs utilizing either VH3-53/3-66 or other VH germline genes are shown in teal and grey, respectively. Statistical comparisons were performed by two-sided Kruskal-Wallis tests with Dunn’s multiple comparisons test. The dotted line indicates the lower limit of detection. Black bars indicate geometric means. (D) Proportion of potently neutralizing mAbs from each donor that utilize either VH3-53/3-66 or other VH germline genes. The number of mAbs analyzed are shown above the bar. (E) Correlation between VSV-SARS-CoV-2 neutralization IC_50_ and SHM load (left) or SARS-CoV-2 S binding affinity (right) for VH3-53/3-66 class mAbs. R^2^ values were generated by linear regression analysis. (F) Fab binding activities of 82 potently neutralizing antibodies (VSV-SARS-CoV-2 IC_50_ < 0.1 μg/ml) to yeast displayed RBDs containing the indicated amino acid substitutions. Amino acid substitutions were derived from emerging SARS-CoV-2 isolates in South Africa (SA, B.1.351/501Y.V2), Brazil (BR, B.1.1.28/501Y.V3), or the United Kingdom (UK, B.1.1.7/501Y.V1). Values indicate the percent Fab binding affinity to the mutant RBD relative to Wuhan-1 (WT) SARS-CoV-2 RBD. Clinical-stage SARS-CoV-2 antibodies are included as controls. K.O. indicates binding below the limit of detection. K.O., knock-out. WT, wild-type. *, P < 0.05; ns, not significant.

Next, we screened the S-reactive mAbs for neutralizing activity against VSV-SARS-CoV-2 at a single IgG concentration of 50 nM (7.5 μg/ml). At the first sampling time point, only a small fraction (0-10%) of mAbs from each donor repertoire showed potent neutralizing activity, defined as >80% inhibition of viral infection at the concentration tested (Fig. 3A and Fig. S5). By Visit 3, the proportion of potent nAbs increased to >10% of the S-specific response in all but one donor (Fig. 3A and Fig. S5). Although the donors with severe COVID-19 displayed more potent serum nAb responses and higher magnitude MBC responses relative to the donors with mild disease (Fig. 1D, E), we did not observe a significantly higher frequency of potent nAbs in their MBC repertoires at the time points studied (Fig. 3A). Thus, the higher serum nAb titers observed in these donors was likely due to a more robust expansion of S-specific B cells rather than repertoire skewing toward neutralizing specificities.

### Recurrent RBD-directed antibodies dominated the neutralizing response to SARS-CoV-2

We next determined the neutralization half-maximal inhibitory concentrations (IC_50_s) of all mAbs that displayed >80% neutralizing activity in the initial screen. Consistent with prior reports (*13*, *21*, *23*, *24*), the vast majority of potently neutralizing mAbs (IC_50_ < 0.1 μg/ml) were directed to the RBD and interfered with hACE2 receptor binding (Fig. 3B). Overall, the nAbs isolated at Visits 2 and 3 trended toward higher neutralization potencies relative to the nAbs from Visit 1, suggesting that improvements in antibody binding affinity translated into enhanced neutralization potency, at least for a proportion of the response (Fig. 3C). For all donors, ≥50% of the highly potent nAbs were encoded by the closely related VH3-53 or VH3-66 germline genes, all of which recognized the RBD and blocked hACE2 binding (Fig. 3D, Table S2, and Table S3). These nAbs also shared multiple common somatic mutations, including F28L/V and Y66F in CDRH1 and CDRH2, respectively (Table S3). Inspection of previously published crystal structures of VH3-53/3-66 class nAbs revealed that these shared SHMs form contacts with the RBD, supporting convergent evolution to facilitate binding to a common antigenic site (*25*–*27*) (Fig. S6A). Notably, the VH3-53/3-66-class (also known as “Class 1” (*26*)) nAbs displayed comparable SHM loads and binding affinities to those observed for nAbs encoded by other VH germline genes, suggesting that their near-universal ability to neutralize is likely linked to their epitope specificity rather than high intrinsic germline-encoded affinity or accelerated affinity maturation (Fig. S6B, C). Furthermore, there was no correlation between neutralization potency and affinity or SHM load for VH3-53/3-66 class nAbs, demonstrating that potent neutralization of SARS-CoV-2 can be achieved by near-germline antibodies and further affinity maturation may not lead to additional improvements in neutralization potency (Fig. 3E).

### Potently neutralizing antibodies displayed reduced activity against emerging SARS-CoV-2 variants

Given the recent emergence of several SARS-CoV-2 genetic variants harboring mutations in the sites recognized by commonly induced SARS-CoV-2 nAbs, including VH3-53/3-66 class nAbs, we evaluated the Fab binding affinities of the 82 most potently neutralizing antibodies in our panel (IC_50_ < 0.1 μg/ml) to yeast surface displayed RBDs derived from the SARS-CoV-2 Wuhan-1 strain or emerging isolates in the United Kingdom (B.1.1.7/501Y.V1), South Africa (B.1.351/501Y.V2) and Brazil (B.1.1.28/501Y.V3) that have been associated with rapidly increasing case numbers (*5*, *7*). The UK isolate contains a single amino acid substitution in the RBD at position 501 (N501Y), whereas the South African and Brazilian isolates encode three mutations at positions 501, 484, and 417 (N501Y, E484K, and K417N in B.1.351/501Y.V2 and N501Y, E484K, and K417T in B.1.1.28/501Y.V3). We also measured the binding affinities of several clinical-stage antibodies as controls. Consistent with prior neutralization studies (*5*, *6*), the clinical-stage antibodies S309 and REGN10987 retained activity against all three variant RBDs, LY-CoV16 displayed reduced binding to all three variant RBDs, and LY-CoV555 and REGN10933 showed substantially diminished activity against the South African and Brazilian isolate RBDs, thus validating the use of RBD binding affinity to predict neutralization escape (Fig. 3F).

Sixty-two percent and 52% of the 82 potently neutralizing antibodies in our panel displayed >10-fold reduced binding affinity to the RBDs derived from the South African and Brazilian isolates relative to the Wuhan-1 (wild-type) RBD, respectively, whereas only 9% (8/82) of nAbs bound with >10-fold reduced activity to the UK isolate-derived RBD (Fig. 3F and Fig. S6D). Hence, it appears that the reduced nAb binding activities against the South African and Brazilian isolates are primarily mediated by the substitutions at positions 484 and/or 417 rather than 501. Notably, these results are consistent with recent studies demonstrating that all three of these emerging SARS-CoV-2 isolates display reduced susceptibility to neutralization by convalescent and vaccinee sera, with SARS-CoV-2 B.1.1.28/501Y.V3 and B.1.351/501Y.V2 exhibiting markedly increased resistance to neutralization relative to B.1.1.7/501Y.V1 (*5*–*7*).

To investigate the relative contributions of the mutations at positions 484 and 417 to the reduced nAb binding affinities to the South African and Brazilian isolate RBDs, we tested the Fabs for reactivity with RBDs containing individual E484K, K417N, or K417T substitutions. The abrogated binding observed for VH3-53/3-66-class nAbs against these isolates was largely mediated by the substitutions at position 417, with the E484K mutation contributing to a lesser extent (Fig. 3F). Within this class of nAbs, susceptibility to these mutations appeared to be partially dependent on VL germline gene usage, with 92% (12/13) of nAbs encoded by VK1-9 exhibiting <10% binding activity to the South African and/or Brazilian isolate RBDs compared to only 14% (1/7) of nAbs utilizing VK3-20 (Table S2, S3). In contrast to VH3-53/3-66-class nAbs, the diminished binding activity observed for nAbs utilizing other VH germline genes was primarily mediated by the E484K substitution (Fig. 3F). Importantly, the sensitivity of these nAbs to E484K suggests that they belong to a second, previously described class of recurrent RBD-directed antibodies, known as “Class 2” nAbs, that utilize a variety of different VH and VL germline gene combinations to recognize a shared epitope that often involves residue 484 (*26*). In summary, the majority of nAbs induced by natural SARS-CoV-2 infection display reduced binding activity to emerging SARS-CoV-2 variants in Brazil and South Africa, thus providing a potential molecular explanation for the reduced susceptibility of these isolates to neutralization by convalescent sera (*5*–*7*).

### Cross-reactivity of the SARS-CoV-2 S-specific memory B cell response

**Fig. 4:**
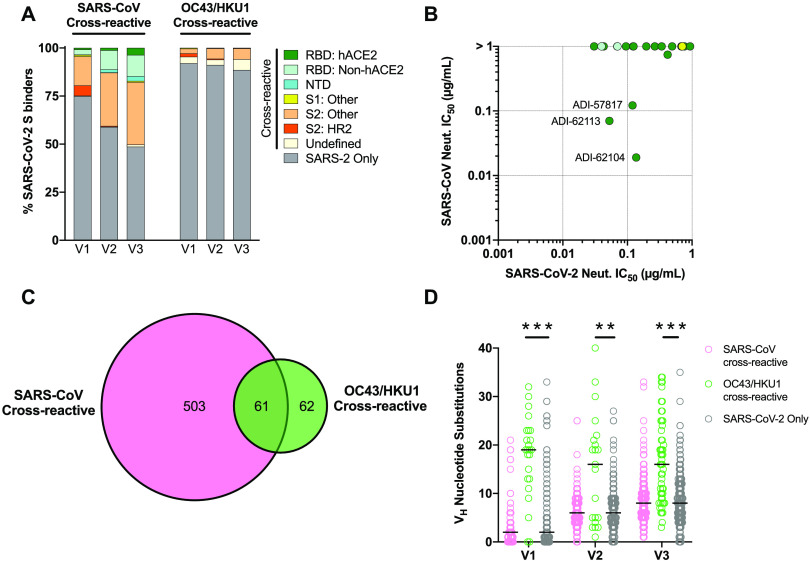
Cross-reactivity properties of anti-SARS-CoV-2 S mAbs. (A) Proportion of SARS-CoV-2 S-specific mAbs isolated at Visits 1-3 that cross-react with the SARS-CoV (left) or endemic β-CoV (right) S proteins, averaged across all donors. MAbs that showed cross-reactivity with OC43 and/or HKU1 S are grouped together and designated OC43/HKU1. The antigenic sites targeted by the cross-reactive mAbs are indicated with colors, as defined in the legend. All SARS-CoV-2 monospecific mAbs are grouped together and shown as a single dark grey segment. (B) VSV-SARS-CoV and VSV-SARS-CoV-2 neutralization IC_50_s for all SARS-CoV cross-reactive mAbs that displayed a SARS-CoV-2 neutralization IC_50_ < 1 μg/ml. Colors correspond to antigenic sites as defined in (A). (C) Venn diagram showing the cross-reactivity profiles of mAbs isolated at Visits 1-3. All time points are pooled for this analysis. (D) SHM loads of cross-reactive and SARS-CoV-2 monospecific mAbs isolated at Visits 1-3. Statistical comparisons were made by two-sided Kruskal-Wallis tests with Dunn’s multiple comparisons test. Black bars indicate medians. **, P < 0.01; ***, P< 0.001.

We next assessed the mAbs for binding cross-reactivity with full-length S proteins derived from SARS-CoV and seasonal β-CoVs (OC43 and HKU1). 11-40% of the mAbs isolated from the Visit 1 samples displayed cross-binding activity to SARS-CoV S, and the proportion of cross-binding antibodies increased over time in all donors (Fig. 4A and Fig. S7A). By Visit 3, 37%-62% of mAbs from each donor showed SARS-CoV S cross-reactivity (Fig. S7A). The cross-reactive mAbs recognized multiple distinct antigenic sites within both the S1 and S2 subunits, with the largest fraction targeting epitopes in S2 outside of HR2 (Fig. 4A). Despite the large number of SARS-CoV/SARS-CoV-2 cross-binding mAbs, only 0.6% (3/540) displayed potent cross-neutralizing activity, all of which targeted epitopes within the RBD and interfered with hACE2 binding (Fig. 4B and Table S2).

In contrast to the high degree of cross-reactivity with SARS-CoV S, only 8-11% of mAbs isolated at the three sampling time points showed binding reactivity with OC43 and/or HKU1 S proteins (Fig. 4A). In most donors, the proportion of seasonal β-CoV cross-reactive mAbs remained stable or increased in frequency over time (Fig. S7B). The majority of these cross-reactive mAbs targeted epitopes within the S2 subunit and about 50% also recognized SARS-CoV S (Fig. 4C and Table S2). However, all OC43 and HKU1 S-reactive mAbs lacked neutralizing activity against both SARS-CoV and SARS-CoV-2, which may be associated with their relatively low binding affinities for SARS-CoV-2 S (Table S2). Importantly, the OC43 and HKU1 cross-reactive mAbs isolated at each sampling time point displayed significantly higher SHM loads than SARS-CoV-2 monospecific mAbs (Fig. 4D), and about half of these cross-reactive B cells expressed the activation marker CD71 (Fig. S7C), providing evidence that pre-existing cross-reactive MBCs induced by prior β-CoV infections are recruited into the SARS-CoV-2 S-specific MBC response.

## DISCUSSION

Longitudinal studies of natural infection provide valuable insights into the kinetics and durability of protective immune responses and can guide rational vaccine design. The results described here illuminated several key features of the MBC response to SARS-CoV-2 infection. First, consistent with recent studies (*9*, *20*, *28*), we observed a significant decline in serum nAb titers over the course of ~120 days, but SARS-CoV-2 S-specific IgG^+^ MBCs remained stable or increased in frequency over this period. Although sterilizing protection can only be achieved by high titers of serum nAbs, MBCs can contribute to protection against clinically significant breakthrough infections. For example, due to the long incubation period and slow progression of hepatitis B virus disease, robust anamnestic responses produced by re-activated MBCs can provide protection against symptomatic disease even in the absence of detectable circulating serum antibodies (*29*). The relatively slow progression to severe COVID-19 suggests that there may be a window of opportunity for MBCs to expand, differentiate, and produce protective levels of serum antibody before the onset of clinical disease (*30*).

A study of *postmortem* spleen and lymph node samples from severe COVID-19 patients revealed striking defects in GC formation, suggesting that SARS-CoV-2 infection may compromise the generation of long-lived plasma cells and MBCs (*31*). In our cohort of COVID-19 survivors, we observed increases in antibody SHM, binding affinity, and neutralization potency for several months following SARS-CoV-2 infection, suggesting that GC activity continues for many months following the period of active viral replication. Two recent longitudinal studies of SARS-CoV-2 infection also reported similar findings (*20*, *28*). Although we cannot exclude the possibility that viable SARS-CoV-2 persists at low levels in tissue reservoirs, previous studies demonstrate that inert viral antigens, in the form of complement-coated immune complexes, can be retained on the surface of follicular dendritic cells for extended periods of time for presentation to GC B cells (*32*). In support of SARS-CoV-2 antigen persistence, a recent study demonstrated that SARS-CoV-2 mRNA and protein are detectable in the gastrointestinal tracts of COVID-19 patients several months following resolution of clinical disease (*28*). Importantly, MBC responses induced by Ebola and yellow fever virus continue to mature for 6-9 months following infection or vaccination, suggesting that prolonged antibody affinity maturation may be a common feature of the B cell response to primary acute viral infection (*18*, *19*).

Unexpectedly, we found that pre-existing, cross-reactive MBCs induced by seasonal β-coronavirus exposures were re-activated by SARS-CoV-2 infection and persisted in peripheral blood for several months following symptom-onset. We also observed elevated serum antibody responses to OC43 and HKU1 at all three timepoints studied, potentially suggesting migration of cross-reactive antibody-secreting cells to the bone marrow and maintenance of these cells in this niche for at least several months. In addition to cross-reactive “re-called” antibodies, a large proportion of the de novo antibody response was directed to conserved epitopes shared between SARS-CoV-2 and SARS-CoV. However, the overall lack of cross-neutralizing activity exhibited by both classes of broad binding antibodies suggests they are unlikely to provide clinically meaningful protection against antigenically drifted SARS-CoV-2 strains or future emerging CoVs. Rather, “pan-CoV” vaccines will likely require epitope-based immunogen design strategies that focus the antibody response on conserved neutralizing epitopes, such as those defined by COVA1-16, ADG-2 and S309 (*33*–*35*).

Finally, the neutralizing response at both early and late time points was dominated by recurrent RBD-directed antibodies that display reduced binding activity against emerging SARS-CoV-2 isolates in Brazil and South Africa that harbor mutations at positions 501, 484, and 417 in the S protein. Previous studies have demonstrated that these nAbs employ common modes of binding and often select for escape mutations at positions 484 and 417 (*25*, *26*, *36*, *37*). The remarkable convergence of SARS-CoV-2 nAb recognition across donor repertoires, combined with the high mutational tolerance of the RBD (*38*), is likely driving the rapid emergence SARS-CoV-2 variants capable of escaping serum neutralizing antibody responses (*5*–*7*). Thus, it will be of critical importance to carefully monitor circulating SARS-CoV-2 isolates for variability in these dominant antigenic sites and determine the impact of these mutations on vaccine-induced immunity. Finally, the results suggest that prime-boost immunization strategies that induce nAbs to several distinct antigenic sites may be required to constrain viral evolution, as recently demonstrated in the context of measles virus vaccination (*39*).

## MATERIALS AND METHODS:

### Study design

A total of eight patients with COVID-19 were recruited for this study. SARS-CoV-2 infection was defined as confirmed reverse transcriptase polymerase chain reaction on nasal swab. Eight donors were included in this study because this was the largest number of donors for which large numbers of antibodies could be practically cloned and characterized. At least two independent experiments were performed for affinity measurements, neutralization assays, and antibody competition assays, and the results shown are derived from representative experiments. The clinical and biological characteristics of the donors are listed in Table S1. All volunteers gave informed consent using a template approved by the Dartmouth-Hitchcock Hospital (D-HH) Human Research Protection Program (Institutional Review Board) with the clinical component coordinated by the Clinical Research Unit of D-HH. This study was unblinded and not randomized.

### Coronavirus antigens

To generate prefusion-stabilized SARS-CoV-2 S-2P spike protein, a plasmid encoding residues 1-1208 of the SARS-CoV-2 spike with a mutated S1/S2 furin cleavage site (RSAR to GSAS), proline substitutions at positions 986 and 987 and a C-terminal T4 fibritin domain, HRV3C cleavage site, 8x HisTag and TwinStrepTagwas transfected into HEK-293 cells (DSMZ, ACC 305) using PEIpro (PolyPlus, Cat#115-100). Kifunensine (5 μM) was added 3 hours after transfection and the expressed protein was harvested from cell supernatants for further purification using Ni Sepharose resin (Cytiva, Cat#17531804) and StrepTactin XT Superflow high-capacity resin (IBA Life Sciences, Cat#24030025). Purified protein was polished by size exclusion chromatography, using a HiLoad 16/600 Superdex 200 pg column (Cytiva, Cat#28989335) and a HiLoad 16/600 column packed with 125mL of Superose 6 resin (Cytiva, Cat#17048901) successively, in PBS.

Plasmids encoding residues 1-1160 of the SARS-CoV-2 spike with a mutated S1/S2 furin cleavage site (RSAR to GSAS), proline substitutions at positions 817, 892, 899, 942, 986 and 987 and a C-terminal T4 fibritin domain, HRV3C cleavage site, 8x HisTag and TwinStrepTag (HexaPro SΔHR2); residues 697-1208 of the SARS-CoV-2 spike with an artificial signal peptide, proline substitutions at positions 817, 892, 899, 942, 986 and 987 and a C-terminal T4 fibritin domain, HRV3C cleavage site, 8x HisTag and TwinStrepTag (HexaPro S2); residues 1-1190 of the SARS-CoV spike with proline substitutions at positions 968 and 969 and a C-terminal T4 fibritin domain, HRV3C cleavage site, 8x HisTag and TwinStrepTag (SARS-CoV S-2P); residues 1-1276 of the HCoV-HKU1 spike with a mutated S1/S2 furin cleavage site (RRKRR to GGSGS), proline substitutions at positions 1067 and 1068 and a C-terminal T4 fibritin domain, HRV3C cleavage site, 8x HisTag and TwinStrepTag (HCoV-HKU1 S-2P); residues 1-1278 of the HCoV-OC43 spike with proline substitutions at positions 1070 and 1071 and a C-terminal T4 fibritin domain, HRV3C cleavage site, 8x HisTag and TwinStrepTag (HCoV-OC43 S-2P); residues 1-615 of human ACE2 with a C-terminal 8XHisTag and TwinStrepTag (hACE2); residues 319-591 of the SARS-CoV-2 spike with a C-terminal HRV3C cleavage site, monomeric Fc-tag and 8x HisTag (SARS-CoV-2 RBD-SD1); residues 1-305 of the SARS-CoV-2 spike with a C-terminal HRV3C cleavage site, monomeric Fc-tag and 8x HisTag (SARS-CoV-2 NTD) were transfected into FreeStyle293F cells using polyethylenimine. After 6 days of expression, cell supernatants were harvested and purified by affinity chromatography. The SARS-CoV-2 RBD-SD1 and NTD were purified using Protein A resin (Pierce). HexaPro SΔHR2, HexaPro S2, SARS-CoV S-2P, HCoV-HKU1 S-2P, HCoV-OC43 S-2P, and hACE2 were purified using StrepTactin resin (IBA). Affinity-purified proteins were then subjected to further purification by size-exclusion chromatography using a buffer composed of 2 mM Tris pH 8.0, 200 mM NaCl and 0.02% NaN3. The SARS-CoV-2 RBD-SD1, NTD and hACE2 were purified using a Superdex 200 Increase column (Cytiva) and HexaPro SΔHR2, HexaPro S2, SARS-CoV S-2P, HCoV-HKU1 S-2P, and HCoV-OC43 S-2P were purified using a Superose 6 Increase column (Cytiva).

The SARS-CoV-2 S1 subunit was purchased from Acro Biosystems (Cat# S1N-C52H3). Non-stabilized OC43 S (Cat# 40607-V08B) and HKU1 S (Cat# 40606-V08B) proteins were purchased from Sino Biological.

### Single B cell sorting

B cells were purified directly from human peripheral blood samples using the EasySep Direct Human B Cell Isolation Kit (Stem Cell Technologies Cat# 19674). Purified B cells were stained using anti-human CD19 (PE-Cy7; Biolegend Cat# 302216), CD3 (PerCP-Cy5.5; Biolegend Cat# 30040), CD8 (PerCP-Cy5.5; Biolegend Cat# 344710), CD14 (PerCP-Cy5.5; Invitrogen Cat# 45-0149-42), CD16 (PerCP-Cy5.5; Biolegend Cat# 360712), IgM (BV711; BD Biosciences Cat# 747877), IgD (BV421; Biolegend Cat# 348226), IgA (AF-488; Abcam Cat# Ab98553), IgG (BV605; BD Biosciences Cat#563246), CD27 (BV510; BD Biosciences Cat# 740167), CD71 (APC-Cy7; Biolegend Cat# 334110), propidium iodide (PI), and a freshly-prepared mixture of PE- and APC-labeled SARS-CoV-2 S-2P protein tetramers (25 nM each). Class-switched B cells, defined as CD19^+^CD3^−^CD8^−^CD14^−^CD16^−^PI^−^IgM^−^IgD^−^ cells, that showed reactivity to both SARS-CoV-2 S-2P tetramers were single-cell index sorted using a BD FACS Aria II Fusion (BD Biosciences) into 96-well polypropylene microplates (Corning Cat# 07-200-95) containing 20 μl /well of lysis buffer [5 μl of 5X first strand SSIV cDNA buffer (Invitrogen Cat # 18090050B), 1.25 μl dithiothreitol (Invitrogen), 0.625 μl of NP-40 (Thermo Scientific Cat# 85124), 0.25 μl RNaseOUT (Invitrogen Cat#10777019), and 12.85 μl dH2O]. Plates were immediately spun down at 1,000 × g for 30 s and stored at -80°C until use. Flow cytometry data was analyzed using FlowJo software.

Antibody-secreting cells (ASCs) were identified by staining B cells with anti-human CD19 (PE-Cy7; Biolegend Cat# 302216), CD3 (PerCP-Cy5.5; Biolegend Cat# 30040), CD8 (PerCP-Cy5.5; Biolegend Cat# 344710), CD14 (PerCP-Cy5.5; Invitrogen Cat# 45-0149-42), CD16 (PerCP-Cy5.5; Biolegend Cat# 360712), CD27 (BV510; BD Biosciences Cat# 740167), CD38 (BV421; Biolegend, Cat#303526), and propidium iodide (PI). ASCs were defined as CD19^+^CD3^−^CD8^−^ CD14^−^CD16^−^PI^−^CD27^+^CD38^+^ cells.

### Amplification and cloning of antibody variable genes

Human antibody variable gene transcripts (VH, Vκ, Vλ) were amplified by reverse transcription polymerase chain reaction (RT-PCR) using SuperScript IV enzyme (Thermo Scientific Cat# 18090050) followed by nested PCR using cocktails of variable region and IgM-, IgD-, IgA- and IgG-specific constant-region primers with HotStarTaq Plus DNA Polymerase (Qiagen Cat# 203646), as previously described (*40*). The primers used in the second round of nested PCR contained 40 base pairs of 5′ and 3′ homology for linearized yeast expression vectors to allow cloning by homologous recombination. Amplified variable gene transcripts were transformed into in *S. cerevisiae* using the lithium acetate method for chemical transformation (*41*). For each transformation reaction, 1x10^6^ yeast cells were mixed and incubated with 240 μl of polyethylene glycol (PEG) 3350 (50% w/v) (Sigma-Aldrich, Cat#202444), 36 μl of 1M lithium acetate (Sigma Aldrich, Cat#517992), 10 μl of denatured salmon sperm DNA (Invitrogen, Cat#15632011), 67 μl sterile water, 200 ng of the digested expression vectors and 10 μl each of unpurified VH and VL PCR products at 42°C for 45 min. Following transformation, the yeast were washed twice with sterile water, resuspended in selective media and plated. Finally, individual yeast colonies were picked for Sanger sequencing.

### Expression and purification of IgGs and Fab fragments

Monoclonal antibodies used for binding experiments, competition assays and neutralization assays were produced as full-length IgG1 proteins in *S. cerevisiae* cultures, as previously described (*40*). Briefly, yeast cultures were incubated in 24 well plates at 30°C and 80% relative humidity with shaking at 650 RPM in Infors Multitron shakers. After 6 days of growth, the culture supernatants were harvested by centrifugation and IgGs were purified by protein A-affinity chromatography. IgGs bound to the agarose were eluted with 200 mM acetic acid with 50 mM NaCl (pH 3.5) and neutralized with 1/8 (v/v) 2 M HEPES (pH 8.0).

To generate Fab fragments, IgGs were digested with papain for 2 hours at 30°C followed by the addition of iodoacetamide to terminate the reaction. The mixtures were passed over protein A agarose to remove Fc fragments and undigested IgG. The flow-through of the protein A resin was passed over either KappaSelect resin (Cytiva, Cat#17545803) or LambdaFabSelect resin (Cytiva, Cat#17548203) for antibodies utilizing the kappa or lambda light chains, respectively. The Fabs captured on the resin surface were eluted using 200 mM acetic acid with 50 mM NaCl (pH 0.52.0) and neutralized with 1/8 (v/v) 2 M HEPES (pH 8.0).

### Biolayer interferometry kinetic measurements

Apparent equilibrium dissociation constant (K_D_^App^) affinities were measured by BLI using a ForteBio Octet HTX instrument (Molecular Devices) as previously described (*40*). All reagents were formulated in PBSF (PBS with 0.1% w/v BSA) and all binding steps were performed at an orbital shaking speed of 1000 rpm and 25°C. To measure IgG binding to recombinant antigens, anti-human IgG (AHC) biosensors (Molecular Devices) were used to capture the IgGs (100 nM, 0.6 – 1.2 nm) and then allowed to stand in PBSF for a minimum of 30 min. For experiments involving Strep-tag-encoding antigens, the IgG-loaded biosensors were incubated in a biocytin solution (100 μM) for 10 min to saturate remaining streptavidin binding sites. After a short (60 s) baseline step in PBSF, the IgG-loaded biosensors were exposed (180 s) to the antigen at 100 nM, then dipped (180 s) into PBSF to measure any dissociation of the antigen from the biosensor surface. For binding responses > 0.1 nm, data were aligned, inter-step corrected (to the association step), and fit to a 1:1 binding model using the ForteBio Data Analysis Software, version 11.1.

To measure the monovalent equilibrium dissociation constant (K_D_) affinities of Fabs, streptavidin biosensors (Molecular Devices) were used to immobilize biotinylated antigens (100 nM, 1.0 – 2.0 nm) and then allowed to stand in PBSF for a minimum of 30 min. After a short (60 s) baseline step in PBSF, the antigen-loaded biosensors were exposed (180 s) to the Fab at 100 nM, then dipped (180 s) into PBSF to measure any dissociation of the Fabs from the biosensor surface. For binding responses > 0.1 nm, data were aligned, interstep corrected (to the association step), and fit to a 1:1 binding model using the ForteBio Data Analysis Software, version 11.1.

### Competition analysis using biolayer interferometry

Competition binding of mAbs to recombinant SARS-CoV-2 RBD with human ACE2 (hACE2) was evaluated using a ForteBio Octet HTX instrument (Molecular Devices) as previously described (*40*). All reagents were formulated in PBSF (PBS with 0.1% w/v BSA) and all binding steps were performed at an orbital shaking speed of 1000 rpm and 25°C. Briefly, IgGs (100 nM) were captured onto anti-human IgG (AHC) biosensors (Molecular Devices) to a sensor response of 1.0 nm - 1.4 nm. An inert IgG (0.5 mg/mL) was used to occupy any remaining binding sites on the biosensor then allowed to equilibrate in PBSF for a minimum of 30 min. To assess any cross interactions between proteins on the sensor surface and the secondary molecules, the loaded and blocked sensors were exposed (90 s) to hACE2 recombinant protein (300 nM) prior to the binning analysis. The biosensors were then subjected to a second short (60 s) baseline step in PBSF, followed by an association step (180 s) to recombinant SARS-CoV-RBD (100 nM) and finally exposed (180 s) to hACE2 (300 nM). The data was y-axis normalized, and interstep corrected using the ForteBio Data Analysis Software version 11.0. Additional binding by the secondary molecule indicates an unoccupied epitope (non-competitor), whereas the absence of additional binding indicates epitope blocking (competitor).

### Cloning and expression of SARS-CoV-2 variants RBD constructs

RBD-SD1 regions (spike residues 319 to 591) of SARS-CoV-2 and emerging SARS-CoV-2 variants (lineages B.1.1.7/ 501Y.V1, B.1.351/501Y.V2, and B.1.1.28/501Y.V3) were expressed via yeast surface display for assessment of antibody binding. DNA sequences encoding SARS-CoV-2 Wuhan-1 (GenBank: MN908947.3), SARS-CoV-2 variants incorporating individual amino acid substitutions (K417N, K417T, E484K, N501Y), or combinations of mutations (K417N:E484K:N501Y, K417T:E484K:N501Y) were obtained as gBlocks (IDT) and cloned into a yeast surface-display vector as previously described (*33*). The vector encodes a hemagglutinin (HA) epitope tag linked via a Gly4Ser linker to the N terminus of the RBD-SD1, which is connected to Aga2p at the C terminus via two consecutive Gly4Ser linkers. Plasmids were then transformed into *S. cerevisiae* (EBY100) cultures using the Frozen-EZ Yeast Transformation II Kit (Zymo Research) according to the manufacturer’s protocol and recovered in selective SDCAA media.

Induction of RBD expression was performed as previously described (*33*). Briefly, fresh yeast cultures were grown in SDCAA media at 30°C and 180 rpm until cultures reached an 0.8-1.0 OD600. Cells were centrifuged and resuspended in SGCAA media followed by incubation for 16-20 hours at 20°C and 200 rpm.

### Fab binding to yeast surface displayed RBD variants

Fabs were titrated on yeast surface displayed RBD variants to determine EC_50_ concentrations. Briefly, induced cells were aliquoted (0.2 OD600 / well) into 96-well plates and washed with PBSF. Cells were then resuspended in 50 μL of Fab solution diluted in PBSF and incubated on ice for 2 hours. Cells were subsequently washed twice with PBSF and labeled with 50 μL of APC-conjugated monoclonal mouse anti-hemagglutinin tag (HA).11 antibody (BioLegend, Cat # 901524), PE-conjugated goat anti-human IgG polyclonal antibodies (Southern Biotech, Cat # 2040-09), and propidium iodide (Invitrogen, Cat #P1304MP) for 15 min on ice. Cells were washed twice with PBSF before analyzing via flow cytometry on a BD FACS Canto II (BD Biosciences). Mean anti-human IgG PE fluorescence signal values were fitted as nonlinear regression curves in GraphPad Prism using the following equation: Y=Y_minimum_+ (X^Slope) * (Y_maximum_-Y_minimum_)/(X^Slope + EC_50_^Slope)), where X is the Fab concentration and Y is the PE MFI. Concentrations displaying hook effects, defined as concentrations higher than those generating the maximum PE MFI signal, were excluded from analysis. The percent reduction in binding activity to the mutant RBD relative to the WT SARS-CoV-2 RBD was calculated using the following equation: (WT EC_50_/Variant EC_50_) * 100.

### VSV-SARS-CoV-2 pseudovirus neutralization assay

Microneutralization assays were performed using a VSV-based pseudovirus system as previously described (*42*, *43*). COVID-19 convalescent sera or mAbs were diluted in 2-fold series and incubated with VSV-SARS-CoV-S or VSV-SARS-CoV-2-S pseudoviruses for 1 hour at 37°C before addition to 293T-hsACE2 cells (Integral Molecular, Philadelphia, PA). The cells were incubated at 37°C, 5% CO_2_ for 24 hours, after which luciferase activity was measured in cell lysates using the Bright-Glo system (Promega) with a Bio-Tek II plate reader. Percent neutralization was calculated as [100 - (mean RLU test wells/mean RLU positive control wells) × 100] and used to determine the 50% neutralization titers for serum (NT_50_) and half-maximal inhibitory concentrations for mAbs (IC_50_).

### Enzyme-linked immunosorbent assays (ELISA)

For serum binding studies, 96-well high-binding polystyrene ELISA plates (Corning, Cat#3690) were coated with 25 μl per well of SARS-CoV-2 S-2P, SARS-CoV-2 NTD, SARS-CoV-2 RBD, OC43 S, or HKU1 S proteins diluted to 5 μg/ml in PBS (pH 7.4) and incubated overnight at 4°C. Wells were washed 3 times with PBS then blocked with 5% (w/v) non-fat dried milk (NFDM) in PBS for 1 hour at 37°C. After removal of the blocking solution, serial dilutions of human serum in 5% NFDM-PBS were added to the wells and allowed to incubate for 1 hour at 37°C. Plates were washed three times with PBS then secondary cross-adsorbed anti-human IgG-HRP (Thermo Fisher Scientific, Cat#31413) detection antibody was added at 1:8000 dilution in 5% NFDM-PBS and incubated for 1 hour at 37°C. After washing three times with PBS, 25 μl per well of 1-Step Ultra TMB-ELISA Substrate Solution (Thermo Fisher Scientific, Cat#34029) was added to detect binding, followed by addition of an equal volume of stop reagent. Absorbance was measured at 450 nm using a Spectramax microplate Reader (Molecular Devices).

For mAb binding studies, 96-well ELISA plates were coated with 25 μl per well of SARS-CoV-2 RBD, NTD, S1 subunit or SARS-CoV S protein diluted to 5 μg/ml in PBS and incubated overnight at 4°C. Wells were washed 3 times with PBS and then blocked with 5% NFDM-PBS for 1 hour at 37°C. After removal of blocking buffer, test mAbs diluted to 100 nM in 5% NFDM-PBS were added and incubated for 1 hour at 37°C. Plates were then washed three times with PBS and then secondary cross-adsorbed anti-human Fab-HRP (Jackson ImmunoResearch, Cat#209-035-097) detection antibody was added at 1:10000 dilution in 5% NFDM-PBS and incubated for 1 hour at 37°C. After washing three times with PBS, 25 μl/well of 1-Step Ultra TMB-ELISA Substrate Solution (Thermo Fisher Scientific, Cat#34029) was added to detect binding followed by addition of an equal volume of stop reagent. Absorbance was measured at 450 nm using a Spectramax microplate Reader (Molecular Devices). IgGs that displayed an ELISA binding OD_450_ > 0.8 were designated as binders.

## Supplementary Materials

immunology.sciencemag.org/cgi/content/full/6/56/eabg6916/DC1 Fig. S1. Representative flow cytometry gating strategies. Fig. S2. Representative FACS gating strategy used for SARS-CoV-2 S-specific B cell sorting. Fig. S3. Index sorting analysis. Fig. S4. SARS-CoV-2 mAb sequencing and binding analysis. Fig. S5. Neutralizing properties of SARS-CoV-2 S-specific mAbs. Fig. S6. Characterization of VH3-53/3-66-class nAbs. Fig. S7. Cross-reactivity of anti-SARS-CoV-2 antibodies. Table S1. Donor characteristics. Table S2. Sequencing, binding, cross-reactivity, and neutralization properties of isolated mAbs. (Excel datasheet) Table S3. Sequence and binding characteristics of potently neutralizing VH3-53/66 germline antibodies. Table S4. Raw data (Excel datasheet)
